# Recent models for adaptive personality differences: a review

**DOI:** 10.1098/rstb.2010.0221

**Published:** 2010-12-27

**Authors:** Niels J. Dingemanse, Max Wolf

**Affiliations:** 1 Department of Behavioural Ecology and Evolutionary Genetics, Max Planck Institute for Ornithology, Seewiesen, Germany; 2 Centre for Adaptive Behaviour and Cognition, Max Planck Institute for Human Development, Lentzeallee 94, 14195 Berlin, Germany

**Keywords:** adaptive individual variation, behavioural syndromes, personality, evolution, model, theory

## Abstract

In this paper we review recent models that provide adaptive explanations for animal personalities: individual differences in behaviour (or suites of correlated behaviours) that are consistent over time or contexts. We start by briefly discussing patterns of variation in behaviour that have been documented in natural populations. In the main part of the paper we discuss models for personality differences that (i) explain animal personalities as adaptive behavioural responses to differences in state, (ii) investigate how feedbacks between state and behaviour can stabilize initial differences among individuals and (iii) provide adaptive explanations for animal personalities that are not based on state differences. Throughout, we focus on two basic questions. First, what is the basic conceptual idea underlying the model? Second, what are the key assumptions and predictions of the model? We conclude by discussing empirical features of personalities that have not yet been addressed by formal modelling. While this paper is primarily intended to guide empiricists through current adaptive theory, thereby stimulating empirical tests of these models, we hope it also inspires theoreticians to address aspects of personalities that have received little attention up to now.

## 1. Introduction

Individuals within single populations often differ consistently in their behavioural tendencies across time and contexts (Wilson 1998; Sih *et al*. 2004*a*,*b*; Réale *et al*. 2007). Male great tits (*Parus major*), for example, differ consistently in whole suites of correlated traits, with more aggressive individuals also tending to be more explorative towards novel objects and unfamiliar environments than less aggressive ones (Verbeek *et al*. 1996). Over the last few years, the notion that such personality types, or behavioural syndromes, exist in a wide range of animal species has stimulated empirical research on the proximate and ultimate factors shaping such variation (Dingemanse & Réale 2005; Sih & Bell 2008; Stamps & Groothuis 2010*a*,*b*). At the same time, researchers have started to develop conceptual frameworks for understanding the basic phenomena associated with animal personalities (Wilson 1998; Dall *et al*. 2004; Sih *et al*. 2004*a*,*b*; Réale *et al*. 2007; Sih & Bell 2008; Wolf & Weissing 2010; Dingemanse *et al*. 2010*b*). In parallel, various theoretical models have been developed to explain and predict particular aspects of animal personalities—these recent models are the focus of this paper.

We start by briefly outlining patterns of individual differences in behaviour that require explanation (§2). We then review formal (and some verbal) models for adaptive personality differences (§3), where we focus on two main questions. First, what is the basic conceptual idea underlying the model? Second, what are the key assumptions and predictions of the model? We conclude by discussing features of consistent individual differences that have not yet been addressed by models (§4). The aim of this review is to guide empiricists through recent models for adaptive animal personalities and stimulate tests of the assumptions and predictions of these models.

## 2. Patterns of individual variation requiring explanation

The empirical literature on animal personality has reported three types of behavioural patterns that require adaptive explanation in the context of animal personality variation (Dall *et al*. 2004; Sih *et al*. 2004*b*; Dingemanse *et al*. 2010*b*). First, consistent individual differences exist in single behaviours. Second, consistent individual differences exist in suites of functionally distinct behaviours. Third, consistent individual differences exist in behavioural plasticity (also called *responsiveness*). In all cases, consistency refers to both stability over time (in terms of date or age) and/or contexts (environmental gradients, e.g. predation risk).

**Figure 1.**
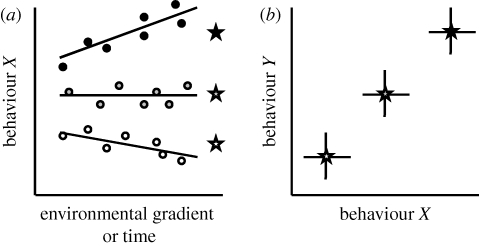
Three types of consistent individual variation in behaviour. Panel (*a*) illustrates the presence of *consistent individual variation in a single behaviour* using a reaction norm plot. Dots represent phenotypic values measured for each of three individuals (black, grey, white) along a contextual gradient (which could be time); lines depict the reaction norm of each individual, and stars give their average phenotype. Individuals differ consistently in average level of behaviour because rank order differences are maintained over an environmental gradient (which could be time in terms of age or date). *Individual variation in plasticity* is also depicted (as individuals differ in reaction norm slope). Panel (*b*) illustrates the presence of *consistent individual variation in suites of behavioural traits*, because individual means (stars) are correlated across behaviours (*X* and *Y*), where vertical and horizontal lines acknowledge the presence of within-individual variation (due to plasticity or measurement error).

Patterns consistent with consistent individual variation in a single behaviour are illustrated in figure 1*a*, which depicts behavioural phenotypes over time (or alternatively an environmental gradient) for each of three individuals (black, grey and white), where lines depict their reaction norms (*sensu* Sarkar 1999). The key feature here is that the rank order differences between individuals are maintained over time or contexts (Sih *et al*. 2004*a*,*b*; Bell 2007).

In many species, individuals differ consistently not only in single behaviours but also these differences involve whole suites of behaviours (van Oers *et al*. 2005; Bell 2007; Réale *et al*. 2007), resulting in correlations across functionally distinct behaviours at the population level (figure 1*b*). In many populations of three-spined stickleback (*Gasterosteus aculeatus*), for example, activity, aggressiveness, exploratory behaviour and boldness are positively correlated across individuals (Huntingford 1976; Bell 2005; Dingemanse *et al*. 2007; Brydges *et al*. 2008). Note that we refer here to correlations between an individual's behavioural mean (illustrated with a star in figure 1*a*) across two or more behavioural traits (figure 1*b*), i.e. the between-individual (as opposed to within-individual) correlations (Dingemanse *et al*. 2010*b*).

Individuals differ not only in their average behaviour but also in their level of behavioural plasticity (responsiveness) (Boyce & Ellis 2005; Nussey *et al*. 2007; Smiseth *et al*. 2008; but see Martin & Réale 2008; Dingemanse *et al*. 2010*b*). This phenomenon is also illustrated in figure 1*a*, which depicts a scenario where individuals differ both in their behavioural mean and in their behavioural plasticity (e.g. black circle individuals are more responsive than grey circle individuals). In laboratory rodents, for example, certain individuals adjust their aggressiveness with social context, whereas others do not (Koolhaas *et al*. 1999; e.g. Fuller *et al*. 2005). Moreover, it has recently been suggested that individual variation in plasticity (also referred to as ‘behavioural flexibility’; Coppens *et al*. 2010) might be correlated across traits, i.e. certain individuals might be consistently more plastic in a variety of functionally distinct behaviours when compared with others (Boyce & Ellis 2005; Sih & Bell 2008), resulting in plasticity syndromes. There is further evidence suggesting that individual variation in plasticity may also covary with mean levels of behaviour (Dingemanse *et al*. 2010*b*) (as in figure 1*a*). In Ural Owls (*Strix uralensis*), for example, mothers that are on average aggressive in nest defence against humans show greater plasticity in aggressiveness when compared with mothers that are less aggressive (Kontiainen *et al*. 2009).

## 3. Models for adaptive personality differences

Here we review formal modelling studies that have explicitly addressed adaptive personality differences, though we have also included two studies (Stamps 2007; Biro & Stamps 2008) that were not based on formal models. Following the classification developed by Wolf & Weissing (2010), these studies were categorized into three non-exclusive types: (i) models that investigate how differences in state give rise to consistent individual differences in (suites of correlated) behaviours, (ii) models that investigate how feedbacks between state and behaviour can stabilize initial differences among individuals over time and (iii) models based on alternative patterns of explanation (i.e. those not based on variation in state).

### (a) Models based on differences in state

State-dependent personality models are centred around the idea that individuals differ in state, where state can be defined broadly as those features of an organism (e.g. morphological, physiological, neurobiological or environmental) that affect the balance between the costs and benefits of its behavioural actions (Houston & McNamara 1999). For a full discussion of state variables in the context of animal personalities see Wolf & Weissing (2010). Consistent differences in state in combination with state-dependent behaviour potentially provide a powerful explanation for adaptive behavioural differences in suites of correlated behaviours, because (i) variation in state can give rise to state-dependent behaviour (condition-dependent behaviour or individual plasticity) and (ii) single states often simultaneously affect behaviour in multiple contexts. This idea underlies several models that investigated how adaptive personality differences can result from individual differences in states (table 1), such as energy reserves (Rands *et al*. 2003; Luttbeg & Sih 2010), body size (McElreath & Strimling 2006), residual reproductive value (RRV, Wolf *et al*. 2007*a*), productivity (Stamps 2007; Biro & Stamps 2008), metabolic rate (Houston 2010) or fighting ability (Botero *et al*. in press). For example, Wolf *et al*. (2007*a*) demonstrated that natural selection should favour individuals possessing low RRV (also termed assets) to act consistently more boldly and aggressively compared to individuals with high RRV, and Stamps (2007) argued that individuals with relatively high growth rates should differ in suites of ‘risky’ behaviours (those behaviours contributing to the trade-off between growth and mortality) compared to individuals with comparatively low growth rates. Table 1 summarizes the basic assumptions and predictions for each of these models.

**Table 1.** Models that investigate how individual differences in state can give rise to adaptive individual differences in behaviour.

state difference	behavioural context and predicted behavioural differences	basic assumptions of model	origin and stability of state differences	reference
energy reserves	*context*: pair of foragers with repeated choice between remaining in a safe refuge and emerging to forage under the risk of predation.	A. foraging in a pair is advantageous (resulting in decreased predation risk or increased energetic gain).	*origin*: stochastic initial differences. *stability*: short-term stability due to positive feedback between foraging behaviour and energy reserves.	Rands *et al*. (2003)
	*predictions*: the individual with lower energy reserves is more willing to take risks (i.e. emerges from refuge first and returns last).			
body size^a^	*context*: foraging context. Individuals can assess environment for imperfect cue that a predator is present. After observing cue, individuals can either forage or run away. *predictions*: three behavioural types that exhibit cross-context correlations: large individuals always forage, intermediate individuals are responsive^b^, small individuals never forage.	A1. individuals have imperfect information about whether or not predators are present	*origin*: not addressed^c^. *stability*: not addressed.	McElreath *et al*. (2006)
		A2. body size affects the chance of being eaten by predator: larger individuals are less likely to be eaten.		
RRV^d^	*context*: risky choices, i.e. behaviours that put the animal's life in danger.	A. fecundity benefits and mortality risk associated with risky choices are identical for individuals with different RRV.	*origin*: frequency-dependent selection^c^.	Wolf *et al*. (2007*a*)
	*predictions*: individuals with higher RRV are less willing to take risk than individuals with lower RRV. These differences correlate across risky contexts (in the model: ‘aggression’ and ‘boldness’ context).		*stability*: stable over time^e^.	
productivity^f,g^	*context*: behaviours related to the acquisition of food resources that increase both productivity and mortality.	A. a trade-off exists between productivity and survival: higher productivity is associated with increased mortality.	*origin*: life-history trade-offs^h^. *stability*: stable since deviations from initial productivity path are costly to the individual.	Stamps (2007); Biro & Stamps (2008)
	*predictions*: productive individuals are more willing to take actions that increase productivity at the cost of increased mortality than individuals with low productivity. These differences are correlated across behaviours that increase both productivity and mortality (e.g. foraging under predation risk, aggressive defence of feeding territories).			
metabolic rate	*context*: foraging behaviour that influences intake rate and mortality.	A. a trade-off exists between energy intake and predation risk: high foraging intensity results in a high intake rate but also a high rate of mortality.	*origin*: coevolution of metabolic rate and foraging intensity.	Houston (2010)
	*predictions*: in certain situations, different combinations of foraging intensity and metabolic rate can have equal fitness.		*stability*: stable over time.	
fighting ability	*context*: agonistic interactions where individuals can, before choosing whether to attack or not, signal their fighting ability to rivals.	A1. individuals have imperfect information about their own fighting ability. A2. costs of signals increase with signal intensity but decrease with fighting ability. A3. genetic mechanism that allows for correlation of sender and receiver behaviour.	*origin*: stochastic differences^c^. *stability*: stable over time^i^.	Botero *et al*. (in press)
	*predictions*: coexistence of behavioural types that differ both in their communication strategies and aggressiveness (i.e. for the same fighting ability, behavioural types show different signalling behaviour and different levels of aggression).			

^a^Authors note that identical results may be obtained for differences in skill, energy reserves, experience and immune condition, and discuss an application of their model to differences in awareness or ability to process cues.

^b^Responsive individuals forage when cue is absent and run away when cue is present.

^c^The origin of the state differences is not important for the predictions of this model, i.e. state differences could arise either stochastically or owing to natural selection (see Wolf & Weissing 2010).

^d^Residual reproductive value. Terms in the literature that are used synonymously: future fitness expectation, assets.

^e^Follow-up work showed that stability depends on whether or not feedbacks between state and behaviour are present and whether these feedbacks act to increase (van Doorn *et al*. 2009) or decrease (Luttbeg & Sih 2010) initial differences in RRV; for a brief discussion of this issue see McElreath *et al*. (2007) and Wolf *et al*. (2007*b*).

^f^Productivity refers to either growth rate or rate of offspring production (Biro & Stamps 2008).

^g^The predictions of this work are not based on formal models but on verbal arguments.

^h^Mangel & Stamps (2001) show how trade-offs between growth and mortality can lead to the maintenance of individual variation in growth rates through nearly equal fitness for individuals growing at different rates.

^i^Each individual has a behavioural reaction norm that is stable over time by assumption.

Explaining personality variation via differences in state leaves us with two basic problems (Wolf & Weissing 2010): first, why should there be variation in state in the first place, i.e. what is the origin of state differences? Second, why should state differences among individuals be stable over time? Some of the models in table 1 do not address the origin of state differences explicitly (McElreath & Strimling 2006; Botero *et al*. in press), while others use stochastic effects acting on states (Rands *et al*. 2003), frequency-dependent selection (Wolf *et al*. 2007*a*) or spatio-temporal forms of selection (Stamps 2007; Biro & Stamps 2008) to explain state differences among individuals (table 1); we believe that future models should explicitly discuss the mechanism maintaining the variation in states that are investigated. We discuss the question of consistency of state differences in §3*b*.

To work out whether a state-dependent model explains behavioural variation associated with personalities in real animals, it would be useful to design experimental studies to test model predictions and assumptions (tables 1 and 2). To the extent that a model deals with behavioural time, a straightforward experimental test would be to manipulate the state variable of interest and investigate whether this manipulation results in the predicted behavioural change (table 1). For instance, based on the verbal model of Stamps (2007), we would predict that food restrictions resulting in decreased growth rates should affect the expression of any behaviour that positively affects growth at the cost of survival—but not other types of behaviour (table 1). Similarly, manipulation of RRV should affect the willingness of individuals to take risky actions (i.e. actions that increase fecundity at the cost of mortality), as predicted by the model of Wolf *et al*. (2007*a*) (table 1). Importantly, we note that none of the models listed in table 1 explicitly predicts whether the link between state and behaviour should be underpinned by phenotypic plasticity (i.e. within genotypes or individuals, environmentally induced changes in state produce changes in behaviour) or by a genetic correlation between state and behaviour (at the population level). Plasticity can involve either an early environmental influence producing individual differences in state with long-lasting effects on behaviour (cf. permanent environment effects), or ongoing and often reversible fluctuations in state over an animal's lifetime. Given that most behavioural studies operate at the latter level, it should be emphasized that if an experimental manipulation of state fails to result in the predicted behavioural change(s), the possibility that a state-behaviour link involves either developmental plasticity or a genetic correlation should be investigated before concluding that the assumptions or predictions of the model were not supported.

**Table 2.** Models that investigate the mutual feedback between state and behaviour.

state	behaviour	feedback and its basic assumptions	references
energy reserves	willingness to emerge from refuge and forage under predation risk	*feedback*: positive feedback stabilizes initial differences for pair of foragers: the individual with lower energy reserves is consistently more willing to take risks (i.e. emerge from refuge first and return last).	Rands *et al*. (2003)
		*basic assumptions*: foraging in a pair is advantageous (resulting in decreased predation risk or increased energetic gain).	
experience with responsive behaviour	responsiveness to environmental stimuli	*feedback*: positive feedback stabilizes initial behavioural differences. Individuals differ consistently in their level of responsiveness.	Wolf *et al*. (2008)
		*basic assumptions*: individuals who were responsive in the past face lower cost (or higher benefits) of being responsive again.	
RRV^a^	willingness to take risks^b^	*feedback*: positive feedback stabilizes initial differences in state and gives rise to consistency in risk-taking behaviour.	van Doorn *et al*. (2009); Luttbeg & Sih (2010)
		*basic assumptions*: trade-off between the immediacy of benefits associated with risky actions and their risk (e.g. risky actions increase current fecundity while less risky actions increase future fecundity) (van Doorn *et al*. 2009).	
		*feedback*: negative feedback erodes initial differences.	
		*basic assumptions*: fecundity benefits associated with risky choices accumulate over time such that risk-taking individuals accumulate assets (Luttbeg & Sih 2010).	
size, energy reserves, condition, vigour	boldness in a foraging context	*feedback*: positive feedback stabilizes initial differences.	Luttbeg & Sih (2010)
		*basic assumptions*: individuals with higher state face lower risk of predation while being bold and bold individuals increase their state relative to less bold individuals.	

^a^Residual reproductive value. Terms in the literature that are used synonymously: future fitness expectation, assets.

^b^Actions that put the animal's life in danger.

In many study systems, experimental approaches might not be feasible. In such cases, initial support for state-dependent models would have to come from studies that investigate the direction, sign and strength of covariation between state variables and behavioural traits within a population. For example, Wilson *et al*. (2010) found—as predicted by Wolf *et al*. (2007*a*)—that (a proxy for) the RRV of individuals was positively correlated with their boldness. Where covariation is found, such studies may provide insight into how the link between state and behaviour was encoded (see above), if repeated measures of state and behaviour can be obtained for individuals within a given population. In this case, intra-individual correlations would imply links encoded at the phenotypic level alone, whereas an inter-individual correlation in the absence of intra-individual correlations might represent a genetically encoded link between state and behaviour. Quantitative genetic approaches can then be used to explore the extent to which the observed phenotypic correlation at the population level is due to underlying genetic versus permanent environmental correlations.

State-dependent personality variation can also be studied by assessing whether the amount of interindividual variation in behaviour can be predicted based on the amount of interindividual variation in state. For example, the extent of individual differentiation in those behaviours that contribute to growth-survival trade-offs in the model of Stamps (2007) should disappear in life-history phases where there is no individual variation in growth rates.

We conclude this section by stressing that, to date, few empirical studies exist that have explicitly tested predictions derived from state-dependent personality models (but see Biro & Stamps 2008; Smith & Blumstein 2008; Harcourt *et al*. 2009; Kobler *et al*. 2009; Réale *et al*. 2009; Wilson *et al*. 2010). Such studies are now needed since a dynamic interaction between theoretical and empirical results provides the key to furthering our understanding of animal personalities.

### (b) Models investigating the feedback between state and behaviour

State variables differ in their stability: some states are *inherently stable*, either because they are very costly (e.g. time-consuming) or even impossible to change, other state variables are much more *labile* (Wolf & Weissing 2010). Interestingly, several of the reviewed models (table 1) use apparently labile states to explain consistent differences in behaviour (e.g. energy reserves, fighting ability). In order for this type of state difference to provide a good explanation for animal personalities, we thus need an explanation for why the differences between individuals in labile states should be stable over time.

Feedback mechanisms between labile states and behaviour might provide such an explanation (Sih & Bell 2008; Luttbeg & Sih 2010; Wolf & Weissing 2010): the state of an individual affects its optimal behaviour, which in turn might feedback on its state. The more experience (state) an individual has with a certain behavioural pattern, for example, the more advantageous it might be to exhibit this pattern, which in turn increases the experience with this behaviour. Such *positive feedbacks* between state and behaviour act to stabilize any initial differences in labile states (e.g. individuals with more experience get even more experienced over time). The idea of positive feedbacks is attractive because even minor initial differences in either state or behaviour can be amplified and stabilized through such feedback. Positive feedbacks thus provide a potentially powerful explanatory framework for animal personalities associated with labile state differences.

It should be stressed that feedbacks between state and behaviour need not always reinforce initial differences: state differences might give rise to behavioural differences, which in turn act to decrease initial differences in state. Individuals with low reserves (state), for example, might show a high foraging intensity to avoid starvation and thus increase their reserves. Such *negative feedbacks* tend to erode initial differences in state.

Several models have addressed the dynamic feedback between labile state and behaviour (table 2), and we here detail the four feedback mechanisms that have been investigated upto now.

#### (i) Feedback between energy reserves and foraging behaviour under predation risk

Individuals with low energy reserves are expected to be bolder in a foraging context than those with higher reserves because they have to avoid the risk of starvation. This relation between energy reserves and boldness involves a negative feedback that erodes initial differences in state (and thus behaviour): individuals with low reserves are bolder than individuals with high reserves; they consequently acquire more reserves, thus leading to the convergence of states and therefore behaviour among individuals over time (Luttbeg & Sih 2010).

However, this need not always be the case. Rands *et al*. 2003 (see Rands *et al*. 2008 for an extended analysis) investigated a scenario where a pair of foragers is repeatedly confronted with the choice between remaining in a safe refuge and emerging to forage under the risk of predation. The authors considered the situation where the two individuals differed initially in energy reserves and showed that—provided that foraging in a pair is advantagous (table 2)—such differences can be stabilized by the feedback between energy reserve and foraging behaviour. The individual with lower energy reserves is consistently willing to take on greater risk (i.e. emerges from refuge first and returns last), yet it will remain in poorer energetic condition. In such a situation, differences in energy reserves and risk-taking behaviour will thus be stable for at least some period of time.

#### (ii) Feedback between performance and experience

Individuals often perform better with increased experience (Rosenzweig & Bennett 1996; Brown & Laland 2003), and processes like learning, training and skill formation often increase the abilities and success of behavioural patterns when repeated. It is relatively easy to envisage that such positive feedbacks between behaviour and experience with that behaviour gives rise to stable behavioural differences among individuals: small initial differences in behaviour give rise to differences in experience with the behaviour, which act to reinforce initial behavioural differences.

While these verbal ideas are well known, they have rarely been investigated in formal models. One recent example is provided by Wolf *et al*. (2008), who focussed on a scenario where individuals could repeatedly choose between a responsive and an unresponsive behavioural tactic (frequency-dependent selection maintained both tactics in the population). In the absence of feedbacks (i.e. where individuals do not perform better with increasing experience), individuals are identical at the evolutionary equilibrium and play the same mixed strategy that randomizes between the two behavioural alternatives. However, whenever experience with one of the tactics decreases the costs (or increases the benefits) of employing this tactic again (i.e. individuals perform better with increasing experience), stable behavioural differences among individuals evolve. While this analysis was performed in the context of responsiveness, it should apply to any choice situation where (i) individuals repeatedly have a choice between two behavioural actions (e.g. hawk versus dove, cooperate versus defect, produce versus scrounge), (ii) the two actions are maintained by frequency-dependent selection in the population and (iii) positive feedback is present.

#### (iii) Feedback between RRV and risk-taking behaviour

The asset-protection principle (Houston & McNamara 1989; Clark 1994) provides a link between the RRV (future fitness expectations, assets) of an individual and its risk-taking behaviour: individuals with low assets have little to lose and should therefore be more willing to take risky actions (i.e. actions that increase their fecundity at the cost of increased mortality) than individuals possessing high assets. Wolf *et al*. (2007*a*) showed that this principle can explain consistent individual differences in suites of risky traits. We should stress that risk-taking here refers to behaviours that put the life of an individual in danger, and not to behaviours that are associated with high variance in outcomes.

At first sight it may seem that asset protection involves a negative feedback that erodes differences in assets over time (McElreath *et al*. 2007; Sih & Bell 2008; Luttbeg & Sih 2010). Individuals with low assets are bolder than individuals with high assets. Consequently, the former acquire more resources than the latter, giving rise to the convergence of assets and thus behaviour over time. Asset protection, however, is not always associated with negative feedbacks (Wolf *et al*. 2007*b*; van Doorn *et al*. 2009). We provide three examples. First, the benefits of everyday risky behaviour may often be small relative to the underlying individual differences in fitness expectations. In such cases, initial differences in assets will not be eroded over time and asset protection predicts stable behavioural differences (Wolf *et al*. 2007*a*,*b*). Second, the benefits associated with risky actions may not directly increase the assets of the acting individual but that of kin members (e.g. risky parental care, risky foraging in cooperative breeding animals; Cant & Field 2001). In such a situation, individuals do not experience a direct increase in RRV when taking a risky action. Consequently, differences in risk-taking behaviour do not affect the underlying differences in assets, and behavioural differences are predicted to be stable over time. Third, whenever there is a trade-off between the immediacy of benefits associated with risky actions and their risk (e.g. risky actions increase current fecundity, while less risky actions increase future fecundity) assets and risk-taking behaviour are coupled by a positive feedback (van Doorn *et al*. 2009): behavioural differences among individuals are predicted to be stable over time and even emerge in cases where differences in fitness expectations are initially absent.

#### (iv) Feedback associated with state-dependent safety

Luttbeg & Sih (2010) considered state variables (e.g. size, energy reserves, condition and vigour) that are characterized by the following two features: (i) individuals with a higher state face a lower predation risk (e.g. are better at fleeing or defending themselves) and (ii) bold individuals increase their state relative to shy individuals. Because high-state individuals face a lower mortality risk when compared with low-state individuals, the former should be bolder under predation risk than the latter. This gives rise to a positive feedback since, by being bold, high-state individuals increase their state even more, relative to low-state conspecifics. Initial individual differences in state are thus predicted to increase over time thereby giving rise to stable behavioural differences in boldness.

### (c) Models that are not based on state differences

Up to now our discussion focussed on models that are based on state differences. However, adaptive personality differences need not always reflect state differences among individuals (Wolf & Weissing 2010). We here detail three types of model that have investigated the emergence of stable individual differences in the absence of state differences.

#### (i) Adaptive variation in responsiveness by frequency-dependent selection

Wolf *et al*. (2008) developed a model to investigate how spatial and temporal variation in the environment can give rise to adaptive individual differences in the responsiveness to environmental stimuli. In this model individuals have a choice between two behavioural options (e.g. a risky versus safe patch). The payoffs associated with these options depend on the current state of the environment, which is changing over time or space. Individuals can be either responsive or unresponsive. Responsive individuals sample their environment for cues about its current state and can therefore show behavioural plasticity, i.e. their behaviour is fine-tuned to the current environmental conditions. In contrast, unresponsive individuals (which do not pay the cost of sampling) do not take such cues into account and exhibit a non-plastic behaviour that is good on average. The authors showed that this basic set-up gives rise to frequency-dependent selection on responsiveness. In short, responsive individuals can exploit environmental opportunities (e.g. switch to the more profitable patch). The benefits that are associated with these opportunities, however, will often decrease with the frequency of individuals that exploit these opportunities (e.g. via density-dependent competition for resources) and thus with the frequency of responsive individuals in the population. The benefits of responsiveness are thus negatively frequency-dependent, which promotes the coexistence of responsive and unresponsive individuals. Interestingly, this modelling framework has recently been applied to explain individual variation in responsiveness in a natural population of pike (*Esox lucius*) (Kobler *et al*. 2009).

#### (ii) Variation, responsiveness and adaptive personality differences

Natural populations typically harbour substantial amounts of variation (e.g. due to mutations). A series of recent models have shown that the amount of variation present in a population may have substantial effects on the expected outcome of evolution (Johnstone 2001; McNamara *et al*. 2004, 2008, 2009; Wolf *et al*. in press). The basic idea is that whenever variation in social contexts is present, responsive (socially aware, eavesdropping) strategies that make their behaviour dependent on certain features of their social partners (e.g. physical features, reputation or behavioural history) may be favoured. The presence of responsive individuals, in turn, often drastically changes the selection pressures for the monitored traits.

This coevolutionary process between responsive strategies and the strategies that are monitored, triggered through some initial variation in the monitored trait, can also give rise to animal personalities. McNamara *et al*. (2009) provide an example in which individuals interact with each other in a trust game. In the absence of variation in trustworthiness, costly sampling (i.e. information acquisition about other individuals) is not beneficial. Whenever there is sufficient variation in trustworthiness, however, samplers are favoured. The presence of samplers, in turn, induces disruptive selection on trustworthiness which gives rise to the coexistence of trustworthy and untrustworthy individuals.

Dall *et al*. (2004) verbally discuss a related scenario. They consider aggressive interactions in a hawk–dove game. In the absence of variation in aggressiveness (i.e. the probability of playing hawk), all individuals should evolve to the same mixed strategy that randomizes between the two behavioural actions (hawk and dove). Whenever sufficient variation among individuals is present, responsive (here: eavesdropping) strategies should be favoured. The presence of responsive strategies, in turn, should favour behaviourally consistent individuals (i.e. individuals that either always play hawk or always play dove). Wolf *et al*. (in press) extended these arguments formally and showed that (i) these processes indeed give rise to polymorphic populations in which individuals are either always responsive, hawks or doves, and (ii) more generally, these results apply to all scenarios that can be represented as matrix games with two pure strategies (e.g. hawk–dove or snowdrift games).

#### (iii) Signalling, communication and adaptive personality differences

Botero *et al*. (in press) developed a model to investigate the joint evolution of signal emission and signal interpretation in the context of aggressive interactions. Individuals differing in quality (fighting ability) can express an ornament potentially reflecting this quality. The relationship between ornament size and quality is a heritable reaction norm (a sender code) that can evolve under the influence of mutation and selection. An individual's fighting strategy is determined by an evolvable receiver code, which specifies the probability of attack in a hawk–dove game as a function of the individual's own quality and the opponent's ornament size. Interestingly, the authors find that in the presence of errors in signal production, a single signalling type (i.e. a combination of a sender strategy and interpretation strategy) did not evolve but, depending upon the magnitude of error in signal production, evolution would favour the stable coexistence of two, three or four signalling types. In other words, the evolved populations were polymorphic and individuals differed systematically in the way they sent signals and interpreted the signals sent by others. These differences in signalling strategy, in turn, gave rise to consistent individual differences in behaviour. For moderate levels of error in signal production, for example, the model predicts the emergence and coexistence of two distinct types: aggressive individuals that have a high probability of producing a large ornament and a tendency to attack, and conservative individuals that produce smaller ornaments and have a lower tendency to attack during fights.

## 4. Discussion

How successful have theoreticians been in explaining and predicting patterns of individual differences in behaviour observed in natural populations? And what is still to be done? In our view, the current set of adaptive models should be regarded as a first step—much remains to be done.

### (a) More states

Our review illustrates that the majority of current explanations for animal personalities are based on state differences among individuals. Several state variables have been investigated (table 1). Many others, however, which might be equally or even more important for understanding animal personalities have received little or no attention from theoreticians up to now. There is, for example, accumulating evidence that behavioural differences are often associated with physiological differences, such as metabolic rate (Careau *et al*. 2008; Millidine *et al*. 2009) and stress responsiveness (Koolhaas *et al*. 1999; Schjolden & Winberg 2007), differences in brain structure (Reddon & Hurd 2009) and cognitive mechanisms such as learning ability (Kotrschal & Taborsky 2010). At present, little is known about how natural selection shapes variation in such states and how such variation might be associated with personalities (but see Houston 2010).

### (b) More traits

The majority of models published to date have focussed on a small number of behavioural traits like aggressiveness and boldness. In fact, all models except those by McNamara *et al*. (2008, 2009) and Wolf *et al*. (2008) address aspects of the aggressiveness–boldness syndrome. While this syndrome appears to be widespread, several other axes of behavioural variation are present in both humans and animals (Pervin & John 1999; Gosling 2001). Important examples include variation in cooperativeness (Schürch *et al*. 2010), responsiveness (Dingemanse *et al*. 2010*b*), diet specialization (Bolnick 2004), parental care (Smiseth *et al*. 2008) and sexual promiscuity (Schuett *et al*. 2010). Those axes have received very little attention by theoreticians up to now.

### (c) More ecology

Empiricists are now starting to understand how key ecological variables (such as habitat stability, predation regime) are related to the presence (or absence) and structure of animal personalities in natural populations (Bell & Sih 2007; Dingemanse *et al*. 2007; Brydges *et al*. 2008; Sinn *et al*. 2010). In contrast, very few models discussed above have provided explanations for spatial or temporal variation in personality structure. One interesting exception is the recent model by Luttbeg & Sih (2010), who aimed to reveal the ecological conditions (dis)favouring animal personality variation, providing a set of predictions regarding when personalities should versus should not be expected to evolve. A pattern in need of explanation, for example, is that environments with higher predation risk appear to favour tighter associations between boldness and aggressiveness when compared to those with lower predation risk (Bell & Sih 2007; Dingemanse *et al*. 2007). One might also investigate systematically which features of environments favour adaptive diversification in states—Barber and Dingemanse (2010) use spatial variation in the presence and diversity of parasites as a worked example. It should be noted, however, that empiricists have only recently started to employ sophisticated statistical techniques that enable detailed insight into how animal personality might differ between populations, years or habitats (Dochtermann & Jenkins 2007; Dingemanse *et al*. 2010*a*), and details of how populations might differ in personality structure therefore largely remain to be unravelled. Nevertheless, we believe that a more systematic investigation of how key ecological conditions affect the presence and the structure of personalities would constitute a necessary next step in understanding animal personalities.

### (d) When heritable?

Animal personality traits can be underpinned by heritable variation (Penke *et al*. 2007; Réale *et al*. 2007; van Oers & Mueller 2010), result from environmental factors (Quinn *et al*. 2009; Oosten *et al*. 2010) or be shaped by interactions between genes and environments (Carere *et al*. 2005; van Oers *et al*. 2005; Dingemanse *et al*. 2009). Most of the models we discussed above do not make specific predictions about the extent to which personalities are shaped by either of these factors, including whether links between state and behaviour are caused by phenotypic plasticity or genetic correlations (§3*a*), and we believe that future research should address this issue more explicitly. Furthermore, data from real animals show that behavioural variation is repeatable only over short time spans in certain species (e.g. Sih *et al*. 2003; Bell & Stamps 2004) but over long time spans in others (e.g. Réale *et al*. 2000; Dingemanse *et al*. 2002). Therefore, it would appear useful for theoreticians to develop adaptive models to address the conditions favouring short- versus long-term consistency, and specify the timescale of their analysis.

### (e) Why variation in plasticity?

Behavioural ecologists have recently discovered that certain classes of individuals are behaviourally more consistent than others, and that such individual variation in consistency is caused by individual differences in their behavioural plasticity (Smiseth *et al*. 2008; Coppens *et al*. 2010; Dingemanse *et al.* 2010*b*; Réale & Dingemanse 2010; Stamps & Groothuis 2010*a*; figure 1*a*), e.g. aggressive types are less plastic than non-aggressive ones (Koolhaas *et al*. 1999). Similar patterns have been observed in humans (Boyce & Ellis 2005; Ellis *et al*. 2006). However, few models have yet addressed individual differences in behavioural plasticity (but see Wolf *et al*. 2008; Botero *et al*. in press). It has been argued recently that because individuals can differ in both their average level of behaviour and behavioural plasticity, it would be useful to apply reaction norm approaches to the study of behaviour (Smiseth *et al*. 2008; Dingemanse *et al*. 2010*b*; Nettle & Penke 2010; Stamps & Groothuis 2010*a*,*b*). This approach considers that behavioural variation comes about because individuals can differ both in their average level of behaviour (the *elevation* of a reaction norm) and level of behavioural plasticity (the *slope* of a reaction norm). Such an approach essentially treats differences between individuals in their behavioural consistency as potentially meaningful (Dingemanse *et al*. 2010*b*), and might ultimately enable us to better understand links between personality and plasticity within a single adaptive framework (see Botero *et al*. (in press) for a model on adaptive personality variation using a reaction norm approach).

### (f) More testing

We think that the time has come for empiricists to start testing the assumptions and predictions derived from adaptive models presented in the literature more explicitly (tables 1 and 2). Such feedbacks between empirical and theoretical approaches are now well on its way and will undoubtedly deepen our understanding of personality variation, exemplified by empirical tests of Rands *et al*. (2003, 2008) models concerning leaders and followers (Harcourt *et al*. 2009), Wolf *et al*.'s (2007*a*,*b*) model on risk-taking behaviour (Réale *et al*. 2009; Wilson *et al*. 2010), Stamps' (2007) model concerning relationships between growth and risky behaviours (Biro & Stamps 2008) and Wolf *et al*.'s (2008) model on individual differences in responsiveness (Kobler *et al*. 2009).

## 5. Conclusions

In this paper, we have reviewed recent models for adaptive personality differences in order to guide empiricists through current adaptive theory. Throughout we have focused on the basic conceptual ideas underlying these models, and their key assumptions and predictions. We argue that empiricists should now start designing studies targeted at testing the assumptions and predictions of existing models. At the same time, there is a need for new theoretical models explaining (i) variation in personality axes other than the aggressiveness–boldness syndrome, (ii) the links between ecological factors (like predation risk) and the presence and structure of personality variation, (iii) individual differences in behavioural plasticity, (iv) heritable versus environmentally determined personality variation and (v) conditions favouring personality variation not associated with variation in states.
